# Parenting Capacity Assessment for the Court in a Multifamily Group Setting

**DOI:** 10.3389/fpsyg.2016.01882

**Published:** 2016-11-30

**Authors:** Roberta Di Pasquale, Andrea Rivolta

**Affiliations:** ^1^Dipartimento di Scienze Umane e Sociali, Università dergli Studi di BergamoBergamo, Italy; ^2^Servizio Tutela MinoriSondrio, Italy

**Keywords:** child maltreatment, parenting capacity, parenting assessment models, multifamily group, multifamily observational setting

## Abstract

Parenting capacity assessment in court evaluations is a particularly complex task, given that it is necessary to consider the vast array of distinct and interrelated aspects and abilities which represent parenting, as well as the elevated number of contextual levels that influence parenting quality. The perspective we want to introduce regards the potentiality of the multifamily group as the elective observational setting in parenting capacity assessment.

## Parenting capacity assessment: difficulties and objectives

Assessing parenting capacity constitutes a crucial task both in the process of child protection in cases of alleged or substantiated parent mistreatment and also regarding decisions relative to child placement in cases of highly conflictual marital separation. According to Budd (2001), several sources of difficulty that specifically characterize parenting capacity assessment are identifiable.

The first source of difficulty regards the lack of universally accepted standards relative to the minimum level for attributing sufficient parenting capacity. The absence of shared references exposes the evaluators to the use of vague and subjective criteria, based on their own experiences and personal assumptions.

The second source of difficulty concerns the typically obligatory nature of the assessment context (in which the parenting capacity assessment takes place), which places parents under high levels of stress linked to the involvement of the courts and to eventual consequences resulting from a negative evaluation. This causes defensive behavior in parents as well as the tendency to alter their usual ways of thinking and their personal conduct for reasons of social acceptability, with inevitable repercussions on the reliability and validity of the gathered information.

The third source of difficulty is related to the shortage of instruments specifically designed to measure parenting capacity, instruments that can't be replaced validly by traditional methods of assessment of psychological functioning (e.g., cognitive and personality tests).

Several authors (Reder, 1991; Budd and Holdsworth, 1996; Azar et al., 1998; Reder et al., 2003; Budd, 2005; Jones, 2010), have given themselves the objectives of standardizing parenting capacity assessment models, with the aim of overcoming these difficulties, making their best attempt to defining more specifically the area to be considered and the most appropriate methodologies to be employed in this task. The aforementioned models indicate various areas of parent functioning that are crucial but also difficult to assess.

Even though the models cited above retain their own specific properties, they all reflect the affirmation of the primary goal of observation of parental behavior and of the parent-child relationship over the most usual practices that favor the use of traditional methods and traditional tools of investigation, for example administering cognitive tests, personality tests etc. to the parents. These tools have less validity and reliability regarding such assessment (Carr et al., 2005), and their results at most have indirect implications about parenting capacity. Therefore these instruments may be efficiently employed in order to integrate the results that emerge from clinical observation, but they cannot replace it.

Although these models differ in identifying the single elements of parenting, they all emphasize the need to consider the different complex facets of parenting. A representative list could include nurturing and protection, warmth and emotional involvement, cognitive and social stimulation, guidance and the limit setting to a child's behavior (Di Pasquale and Rivolta, 2013). All these aspects are necessary to support a harmonious development of a child's personality and should be, at most, assessed by the direct observation of parents' behavior while they interact with their child.

All these models underline the importance that a parent possesses and exercises sufficient ability to reflect on his or her interaction with the child in order to recognize and adequately represent the child's point of view and emotional experience—separating them from his or hers—and to intervene with parenting strategies that seem the most appropriate to the mental structure of the child in that situation, eventually adjusting ways of parenting that the parent understands to be insensitive and/or inefficient.

Finally, in all the models cited above it is recognized that an effective parenting capacity assessment cannot ignore the environment of gradually wider relational systems that influence—sometimes in a dramatically powerful way—the parenting style exhibited by a single parent. Among these relational systems we can mention psychological characteristics and temporary mental states of the parent and child, the particularities of their dyadic interaction patterns, the influence of marital relationship, co-parenting and family system, up to and including the quality of the support network which the family can rely on within its community.

It's easy to understand how, for different reasons, the above prerogatives are particularly difficult to satisfy in traditional settings (sessions of 1–2 h in therapy rooms and with limited observation in artificial contexts) and with methods traditionally employed to assess parent capacity. To be optimally carried out, assessments would require prolonged observation of the different aspects of parenting in multiple relational contexts that can affect it, such as the presence of the other parent and/or other children, the pressure produced by stimuli and concurrent environmental demands.

From a few field studies (Budd et al., 2001), it has been shown that often assessment practices used for drafting reports sent to the courts don't conform to the standards dictated by these guidelines. In particular regarding the lack, if not the total absence of naturalistic observations of parent-child interactions.

The perspective we want to introduce regards the potentiality of the multifamily group as the elective observational setting in parenting capacity assessment. We will argue that the multifamily group offers the unique opportunity to directly assess parenting capacity and the parent-child relationship, through prolonged observation and in an almost natural environment, as indicated as highly desirable by the models cited.

## Multifamily therapy

The therapeutic use of the multifamily group began in the 1960s, initially with families of schizophrenic in-patients (Laqueur et al., 1964), and it is now employed for the treatment of different mental disorders such as schizophrenia, (McFarlane et al., 1995; McFarlane, 2002); major depression (Lemmens et al., 2009), eating disorders (Dare and Eisler, 2000; Scholz Asen and Asen, 2001) and relational problems, including intra-family mistreatment. The main feature of this kind of therapy consists of involving some families (containing one or more members who suffer from mental disorders or conditions of psychological distress) in therapeutic group work. The underlying assumption is that in this specific context, families can learn from each other, favoring the emergence of new points of view and new ways to address their problems.

## The multifamily group with mistreating families

A specific multifamily therapy model, which was originally developed at Marlborough Family Service (Asen et al., 1982; Cooklin et al., 1983), deserves particular mention regarding multi-problem families, in which mistreatment of children is a common problem (Asen and Scholz, 2010).

According to this model, multi-family therapy can be defined as a blend of a systemic-family approach and of group therapy involving a variable number of families participating in therapy sessions characterized by various therapeutic group activities. Families who share similar problems are put together in order to jointly participate in the therapeutic intervention. Therefore, each family can benefit from the presence, experience and support of other families and constitute, in turn, a resource for them.

The prototype setting provides an environment similar to the domestic one, suitable to comfortably accommodate more than one family and includes the possibility to prepare and eat meals together. This setting is equipped with video-recording and video-projecting devices in order to allow video-feedback activities. An integral part of this approach is the expansion of the setting to external environments such as a playground, supermarket etc. This model provides an intensive intervention, given that sessions usually last the whole day, sometimes consecutive days, in order to catalyze change through the so-called “greenhouse effect.”

The therapeutic approach is characterized by the continuous change of the intervention format (in an extended multifamily group, in homogeneous subgroups based on sex or age, or based on some other clinically relevant criteria such as in single nuclear families), as well as characterized by the alternation and synergic combination of activities that are practical and experiential in nature -which partially evoke Minuchin's structural approach (Minuchin and Fishman, 1981)—and those that are reflective in nature, partially inspired by systemic practices such as the reflective team (Andersen, 1987).

This model especially emphasizes the therapeutic aspects of the multifamily group, aspects tied to the possibility of facilitating a positive change in single families learning from and helping each other. However, a multifamily intervention with parents who mistreat their children is also used to assess the parents' ability and willingness to modify their own behavior into a more adequate parenting style (Asen, 2007). The multifamily intervention is particularly used in chronic multi-problem families in which severe lacks of adequate child care have been verified, in which multifamily therapy is considered a last resort, as an intensive intervention to mobilize possible resources not found in more traditional approaches. Therefore it is an intervention whose goal is to assess parenting but in a rather specific way relative to the possibility of modifying previously identified abusive behavior.

## Multifamily group as setting for assessing parenting capacity

Here we intend to specifically underline the multifamily group potential in the process of assessing parenting skills, especially in those families who are requested to be evaluated by the courts and whose parenting skills need to be extensively investigated.

Indeed, due to the way it is structured, the multifamily setting permits the unique opportunity of the integrated observation of many of the dimensions of which a parenting style is composed, as well as of some crucial contextual levels that influence parenting quality.

## Multifamily setting's potentiality: places/times, group/people, activities

We are of the opinion that when reflecting on the potentiality of multifamily therapy in assessing parenting capacity, it is useful to focus on three different features of the multifamily setting separately.

The first feature concerns the potentialities linked to the times and places that usually characterize multifamily work. Since it's oriented to provide an almost naturalistic household interactional context, the multifamily setting provides the unique opportunity to assess parenting capacity and the quality of the parent-child relationship in a broad spectrum of situations that would hardly be observable in traditional settings. This organization of time and place, in fact, allows participants to familiarize themselves with the environment, to feel less uncomfortable and to act in a more natural way. At the same time it is particularly appropriate for the stimulation of relational dynamics difficult to observe in more formal, temporary limited and rigidly structured contexts.

In particular, it's possible to verify how long a parent takes care of his or her child as well as the quality of parent supervision and control over a long period of time, in relation to concomitant needs such as meal preparation and in the presence of distracting social stimuli such as informal conversation with other adult group participants. If both parents are present, this structure/arrangement also permits the observation of co-parenting quality, analyzing in what way and with what degree of functionality, parents organize themselves in order to share parenting tasks.

The second feature is specifically related to the simultaneous presence of several families. The fact that therapists' attention is distributed over all participants helps each parent to reduce his or her perception of being in a judgmental and intrusive context, thereby limiting defensive behavior and favoring a more cooperative attitude to the assessment process as well as a greater willingness to constructively accept feedback about his or her parenting qualities.

In addition, the specific composition of multifamily groups allows us to observe child behavior when he or she interacts with peers and adults, permitting a precious direct evaluation of his or her cognitive, emotional and social skills, as well as of the eventual presence of internalizing and externalizing behavior disorders. It also offers the unique opportunity to observe the mediator, facilitator and supervision role carried out by the parent when his or her child interacts with other group participants. For example, does the parent encourage the child to join in with peers or pose a number of obstacles to his or her participation? Does the parent participate constructively in mediating possible conflicts between children, appear indifferent or intervene and discipline the child in insensitive ways?

Another potentiality of the multifamily setting—linked to the presence of multiple families—is that it allows clinicians to appreciate two important assessment elements: the single parent's relational mode both in informal and structured interactions with other children (surrogate parenting) as well as the way he or she interacts with other adults, with particular reference to his or her peer collaboration skills in structured group work. The first element allows us to assess the parent's potentiality of interaction with several children who aren't his or her own, an important aspect in understanding to what extent the eventual parent's dysfunctions are specifically conditioned by the nature of the established relationship between parent and child. The second element provides ecologically valid indications about the single parent's and single families' ability to establish relationships based on respect, listening, cooperation and solidarity with other adults. This constitutes an important indicator of parental social skills and, in particular, of parental ability to draw upon and mobilize forms of mutual help and social support, elements representing a crucial protective buffer for maintaining adequate parenting abilities in the presence of a variety of stressors.

The third feature linked to the use of the multifamily setting in parenting capacity assessment is related to the nature and the typical but obviously not exclusive way of organizing activities. In fact, it's based on the alternation and synergistic combination of practical-experimental activities (e.g., play activities, role playing, group work) and reflective activities (e.g., observation, video-feedback, group discussion on previous activities). This kind of organization of activities is particularly appropriate for stimulating and assessing a parent's ability to reflect on his or her interaction with the child and on the underlying mental experience, even with the help of feedback provided form other parents. Such a reflective process is also favored by the fact that each parent can be given the role to observe the interactive behavior of other participants - including his or her own child with other parents. Such a decentralized position is useful to the parent in order to understand experiences and emotional states that would be more difficult to understand if directly involved in the interaction.

Moreover, as we mentioned before, a sufficient degree of understanding of the emotional experience inside the parent-child relationship is considered a crucial requirement for adequate parenting in parenting capacity assessment models.

## Conclusion

The use of the multifamily setting, though it seems to require substantial use of professional and organizational resources, can be a particularly effective method to carefully carry out a crucial task such as parenting capacity assessment, a task according to which decisions with remarkable impact on families and community will be made.

## Author contributions

RD and AR contributed equally to the conception ad drafting of the work.

### Conflict of interest statement

The reviewer DP declared a shared affiliation, though no other collaboration, with one of the authors RD to the handling Editor, who ensured that the process nevertheless met the standards of a fair and objective review. The other authors declare that the study was conducted in the absence of any commercial or financial relationships that could be construed as a potential conflict of interest.
